# Supplementary material for: A country-wide survey of knowledge and practice regarding prevention, snake identification and pre-hospital management of children with snakebites amongst Public Health Midwives in Sri Lanka
Source: PLoS Negl Trop Dis. 2026 Mar 16;20(3):e0014083. doi: 10.1371/journal.pntd.0014083 (PMC12991233; doi:10.1371/journal.pntd.0014083)
Supplement: Attachment — Submitted filename: Authors response.docx [file pntd.0014083.s002.docx]

**Authors’ response to reviewers’’ comments**

Dear editor and the reviewers,

We would like to extend our heartfelt thanks to the reviewers for their time, effort, and invaluable feedback in reviewing this manuscript. Your insightful comments and constructive suggestions have significantly enhanced the quality of this work. We greatly appreciate your expertise and dedication, which have contributed to refining our study. Your support in the review process is deeply appreciated, and we are grateful for your commitment to advancing research in this important area.

Kavinda Dayasiri

Corresponding author

Please see the responses below for specific comments.

Reviewers' Comments:

Reviewer #2: -

-Were correct statistical analysis used to support conclusions? Yes but further explanation f qualitative methods needed, -Are the results clearly and completely presented? Yes but need direct quotes or more evidence for the qualitative findings

Thank you very much for this important comment. The current study was designed as a quantitative cross-sectional survey, and appropriate statistical analyses were used to support the conclusions presented. No qualitative component was included in this study, and therefore qualitative analytical methods were not performed.

We agree that qualitative exploration of perceptions and contextual factors is valuable; this aspect has been addressed separately in a different study conducted by our team, which specifically focused on in-depth qualitative assessment of related themes. To maintain methodological clarity and focus, the present manuscript reports only the quantitative findings.

Reviewer #3: A sample size calculation was not included. Although the size attained seemed reasonable.

Thank you very much for this observation. A sample size calculation was in fact performed and is described in the Methods section under participant recruitment and selection. The minimum required sample size was calculated using the standard formula n = z²p(1–p)/d², assuming a 95% confidence level, 5% precision, and an expected adequate knowledge proportion of 50% to ensure maximum sample size. After adjusting for a 20% non-response rate, the minimum required sample was 460 participants.

The final sample (n = 1,706) substantially exceeded this requirement, thereby improving the precision and representativeness of the findings. We will revise the manuscript to make this calculation more prominent and clearer for readers.

Reviewer #3: The analysis presented matches the plan and the results in the prose are clearly stated. The tables however are not very clear, specifllcally table 2. This has the question in the questionaire with the answer in bracket. It is difficult to follow. Perhaps sumarizing this as a figure with the actions instead being the subject of the question rather than the entire question.. to make this clearer to the reader. There are 8 tables which are cumbersome to read and could be converted to figures for ease of understanding

Thank you very much for this valuable suggestion. We agree that the presentation of results, particularly in Table 2, could be clearer. The current format lists the original questionnaire items with the expected correct response in brackets, which may reduce readability.

In response, we have revised Table 2 so that first-aid actions are presented as the main items (e.g., Cutting the bite site, sucking venom, Immobilisation, Herbal applications), with columns indicating the correct responses and the proportions of PHMs who identified each action as appropriate or inappropriate. This will make the clinical meaning of the findings clearer and easier to interpret.

Thank you very much for the helpful suggestion regarding converting tables into figures to improve readability. We agree that figures can enhance visual interpretation of key findings. However, we have retained the tables in their revised, simplified format to ensure precise and detailed presentation of the data. Given the descriptive nature of this nationwide survey, we hope that tabulated values allow readers to accurately interpret proportions and make comparisons without loss of numerical detail.

We have reduced the number of tables by converting Table 4 in the initial draft to Figure 1 in the revised manuscript.

We also have removed Table 1 and the information is presented as a paragraph.

Summary and General Comments

Reviewer #1: 1. This study utilized an online questionnaire that respondents completed themselves. The data may be subject to social desirability bias, despite the authors' efforts to minimize it. Additionally, the study does not measure actual practices in the field.

Thank you very much. We have revised the limitations paragraph as advised.

“This study has several limitations. First, the cross-sectional design provides a snapshot of knowledge and practices at a single point in time and does not allow for causal inferences regarding factors associated with knowledge levels. Second, data were collected using a self-administered online questionnaire, and responses may therefore be subject to recall bias and social desirability bias, despite efforts to ensure anonymity and minimize response pressure. Participants may have reported what they perceived to be desirable or expected answers rather than their true beliefs or intended actions.

Furthermore, the findings reflect self-reported knowledge and stated practices rather than direct observation of real-world behaviour. The study did not assess actual first-aid practices performed in field settings, and therefore discrepancies may exist between reported knowledge and real-life responses during snakebite emergencies. Although stratified sampling ensured representation from all districts, participation required access to digital communication platforms; PHMs with limited internet or smartphone access may have been underrepresented. Despite these limitations, the large sample size and nationwide coverage enhance the reliability and generalizability of the findings.”

2. The conclusions drawn are strong, but the suggestions for "structured training" and "curriculum integration" are quite typical. The Discussion section could explore more innovative, system-level, or technology-based solutions to the ongoing challenges noted, such as strong traditional beliefs and transportation issues.

Thank you very much for the suggestion. We have expanded the discussion section by adding the following information. See paragraph 4 in Discussion section.

“Beyond conventional training programmes, addressing the gaps identified in this study requires innovative, system-level, and technology-supported approaches. Given PHMs’ widespread use of mobile phones, integrating snakebite decision-support tools into existing digital health platforms could provide real-time guidance on first aid, danger signs, and referral pathways. Mobile-based visual aids and short video modules in local languages could help PHMs deliver consistent, culturally sensitive education that counters harmful traditional practices without dismissing community beliefs. Establishing a rapid-access teleconsultation or poison information helpline specifically accessible to frontline community health workers may further strengthen early decision-making in remote settings. In parallel, collaboration with local community leaders, school systems, and agricultural networks could support community-wide risk communication campaigns that align biomedical advice with local contexts.”

3. The term "adequate knowledge" is defined as being in the 75th percentile of the composite score, which is a somewhat technical measure. Providing the raw score range or more context in the Results section could help non-technical readers understand this better.

Thank you very much for this comment. We have defined this for each domain; first-aid, snake recognition and overall knowledge along with respective table legends for Tables 2,3 and 5.

4. It is suggested to refer from the paper: "Blockchain-Enabled Healthcare Optimization: Enhancing Security and Decision-Making Using the Mother Optimization Algorithm". This reference discusses blockchain in healthcare and improved decision-making. It supports the need for better awareness of national guidelines and emergency contact services by suggesting a secure and efficient data-sharing platform for public health management Suggested insertion: Conclusion/Future Work

Thank you very much for this interesting suggestion. We agree that secure and efficient digital systems have growing relevance in strengthening public health decision-making and information sharing. While our study focuses primarily on knowledge gaps and training needs among frontline community health workers, we have expanded the Conclusion to acknowledge the potential future role of secure digital health platforms in improving access to national guidelines, emergency contact services, and referral coordination. This addition situates our recommendations within broader developments in digital health systems while maintaining the study’s focus on community-level capacity building.

We have added the following to the Conclusions section.

“Looking ahead, strengthening community-level snakebite management could also benefit from integration with emerging digital health systems that enable secure information sharing, rapid access to national guidelines, and improved coordination between frontline workers and referral centres. Aligning PHM training with such evolving digital public health infrastructure may further enhance timely decision-making and continuity of care, particularly in geographically remote settings.”

The suggested reference was also added, see reference 17.

5. The Discussion acknowledges barriers such as transportation challenges and traditional beliefs. It would be helpful to connect these barriers to technology-driven solutions suggested in other references, such as mobile education tools, visual aids, and secure data sharing. This would change the recommendation from general "training" to "structured training with technology and community involvement".

Thank you for this insightful suggestion. We agree that linking the identified community and system-level barriers with practical, technology-enabled solutions strengthens the implications of our findings. We have revised the Discussion to expand our recommendations beyond general training to include structured, technology-supported capacity-building approaches. Specifically, we now highlight the potential role of mobile-based education tools, visual decision aids in local languages, and integration with digital health and poison information services to support PHMs in real-time. We also emphasize the importance of combining these approaches with community engagement strategies to address deeply rooted traditional beliefs. These additions provide a more actionable and systems-oriented framework for strengthening pre-hospital paediatric snakebite management.

We have added the following paragraph in Discussion and supported with references

“Importantly, these capacity-building efforts should be designed to directly address the structural and sociocultural barriers identified by PHMs. In settings where transport delays and limited facility access hinder timely care, decision-support resources that function at community level can help PHMs prioritise early immobilisation, rapid referral, and clear communication with families. At the same time, educational strategies must acknowledge the strong influence of traditional beliefs reported by participants. Combining structured training with culturally sensitive communication approaches, supported by locally relevant visual materials, may help PHMs counter harmful practices without alienating communities. Embedding these approaches within existing maternal and child health outreach activities would allow snakebite education to become a routine part of community engagement rather than a standalone intervention.”

6. The Discussion section successfully explains the results and places them within the context of other countries facing similar challenges.

Thank you for the comment.

7. The point about cognitive dissonance, where knowledge clashes with harmful practices, is noteworthy and deserves further exploration. This could suggest the need for a behavioral or psychological aspect in the required training.

Thank you for this thoughtful observation. We agree that the coexistence of correct knowledge and endorsement of harmful practices reflects an important behavioural dimension that warrants attention. We have expanded the Discussion to highlight the role of behavioural and psychological factors in influencing first-aid practices, and we now recommend that future training programmes incorporate behaviour change and communication strategies, rather than focusing solely on knowledge transfer.

The following paragraph was added in Discussion section “Behavioural and psychological factors—such as risk perception, social norms, cultural beliefs, and confidence in challenging traditional advice—likely influence how PHMs translate knowledge into action. Training programmes should therefore incorporate behaviour change communication techniques, including scenario-based learning, role-play, and strategies for addressing misconceptions in culturally sensitive ways. Strengthening PHMs’ skills in persuasive health communication and community engagement may help bridge the gap between awareness and practice, ensuring that evidence-based first-aid measures are consistently promoted and adopted at household level.”

Comments

1. Although the sample size is large, perhaps having a justification of the sample size with a power calculation

Thank you for this important suggestion.

We have added the power calculations and updated the methods sections as follows.

“The minimum sample size was calculated using the formula $n=Z^{2}\text{ }p(1-p)/d^{2}$, assuming a 95% confidence level (Z = 1.96), 5% precision (d = 0.05), and an expected proportion of adequate knowledge of 50% to maximize the sample. Accounting for a 20% non-response rate, a minimum of 460 participants was required, providing over 80% power to detect differences in knowledge levels among Public Health Midwives. The study however, achieved a high national response rate, with a considerably larger number of PHMs (n=1706) participating across all districts, enhancing the precision and external validity of the findings.”

2. Tables are difficult to follow. Suggest editing these and/or changing a few to figures to enhance readibility. Specifllcally tables 2 and 6. These have the questions used in the questionnaire with the answer in brackets. It is difficult to follow. Perhaps sumarizing this as a figure with the actions instead being the subject of the question rather than the entire question.. to make this clearer to the reader.

Thank you very much for this valuable comment. We have edited both tables to present findings more clearly. Brackets were removed and correct options were presented separately an in a reader-friendly manner.

In addition, Table 4 is now presented as a Figure to reduce the number of tables in the manuscript.

Table 1 is removed and the information is presented as a paragraph.

3. There are a few grammatical errors that need correction

Thank you. We went through the manuscript carefully and corrected the occasional grammatical errors.

4. For table 3: Chi square test is a measure of association, therefore the term "Deteminant" may not be an accurate term, rather associations in the bivariate analysis

Thank you very much for the comment. This is now updated as “associations” for Tables 2, 3 and 5 including respective subheadings.

Figure resubmission:

While revising your submission, we strongly recommend that you use PLOS’s NAAS tool (https://ngplosjournals.pagemajik.ai/artanalysis) to test your figure files. NAAS can convert your figure files to the TIFF file type and meet basic requirements (such as print size, resolution), or provide you with a report on issues that do not meet our requirements and that NAAS cannot fix.

Thank you. Figure 1 is uploaded separately as a TIFF file.

Editorial comments

1) Please ensure that the CRediT author contributions listed for every co-author are completed accurately and in full.

At this stage, the following Authors/Authors require contributions: Kavinda Chandimal Dayasiri. Please ensure that the full contributions of each author are acknowledged in the "Add/Edit/Remove Authors" section of our submission form.

The list of CRediT author contributions may be found here: <https://journals.plos.org/plosntds/s/authorship#loc-author-contributions>

Thank you very much. Author contributions for Kavinda Dayasiri are updated as advised. Kavinda Chandimal Dayasiri is the same person as Kavinda Dayasiri.

2) Some material included in your submission may be copyrighted. According to PLOSu2019s copyright policy, authors who use figures or other material (e.g., graphics, clipart, maps) from another author or copyright holder must demonstrate or obtain permission to publish this material under the Creative Commons Attribution 4.0 International (CC BY 4.0) License used by PLOS journals. Please closely review the details of PLOSu2019s copyright requirements here: PLOS Licenses and Copyright. If you need to request permissions from a copyright holder, you may use PLOS's Copyright Content Permission form.

Please respond directly to this email and provide any known details concerning your material's license terms and permissions required for reuse, even if you have not yet obtained copyright permissions or are unsure of your material's copyright compatibility. Once you have responded and addressed all other outstanding technical requirements, you may resubmit your manuscript within Editorial Manager.

Potential Copyright Issues:

i) Please confirm (a) that you are the photographer of S1A-S10A, or (b) provide written permission from the photographer to publish the photo(s) under our CC BY 4.0 license.

We confirm that the images included in S1A–S10A were sourced from multiple publicly available internet sources for the purpose of the study and to facilitate accurate assessment and data collection. We did not obtain formal written permissions for each of these images, as some image sources/ potential copyright holders are not traceable or available online.

We understand the copyright requirements of PLOS and, if deemed appropriate by the editorial team, we consent to remove these images from the published version. We would like to clarify that the images were used solely as illustrative material to support the accuracy of data collection and were not intended for commercial use.

Please let us know if any further action is required from our side to comply with PLOS’s CC BY 4.0 licensing requirements.

3) Please amend your detailed Financial Disclosure statement. This is published with the article. It must therefore be completed in full sentences and contain the exact wording you wish to be published.

1) State what role the funders took in the study. If the funders had no role in your study, please state: "The funders had no role in study design, data collection and analysis, decision to publish, or preparation of the manuscript."

Thank you very much. We have updated the Financial Disclosure statement as advised.

2) If any authors received a salary from any of your funders, please state which authors and which funders..

If you did not receive any funding for this study, please simply state: u201c The authors received no specific funding for this work.u201d

Tharuka Perera received a salary under the grant acknowledged in Funding statement. This is duly mentioned under the Financial Disclosure statement.

The updated section is as follows.

“Tharuka Perera received salary support from the University of Kelaniya Research Council Grant (RC/2025/PPCR02) acknowledged in the Funding statement. The funders had no role in study design, data collection and analysis, decision to publish, or preparation of the manuscript.”
